# High γ-Radiation Sensitivity Is Associated with Increased Gastric Cancer Risk in a Chinese Han Population: A Case-Control Analysis

**DOI:** 10.1371/journal.pone.0043625

**Published:** 2012-08-22

**Authors:** Honglin Dong, Xiaowei Jin, Jie Hu, Haifeng Li, Xianli He, Xiaonan Liu, Guoqiang Bao

**Affiliations:** 1 Department of General Surgery, Second Hospital, Shanxi Medical University. Taiyuan, China; 2 Department of Digestive Diseases, General Hospital of Air Forces of PLA, Beijing, China; 3 Department of General Surgery, Tangdu Hospital, The Fourth Military Medical University, Xi’an, China; 4 Xijing Hospital of Digestive Disease, The Fourth Military Medical University, Xi'an, China; National Taiwan University, Taiwan

## Abstract

Hypersensitivity to radiation exposure has been suggested to be a risk factor for the development of several malignancies, but not including gastric cancer. In this case-control study, radiation sensitivity as measured by chromatid breaks per cell (b/c) was examined in cultured peripheral blood lymphocytes (PBLs) from 517 patients with gastric cancer and 525 healthy controls. Our results showed that b/c values were significantly higher in cases than in controls (Mean [SD], 0.47 [0.20] *vs.* 0.34 [0.17]; *P*<0.001). Using the 50^th^ percentile value for controls (0.34 b/c) as the cutoff point, unconditional logistic regression analysis revealed that γ–radiation-sensitive individuals were at significantly higher risk for gastric cancer (adjusted odds ratio [OR] 2.01, 95% confidence interval [CI] 1.49–3.13). Quartile stratification analysis indicated a dose-response relationship between γ-radiation sensitivity and gastric cancer risk (*P* for trend <0.001). When using the subjects in first quartile of b/c values as reference, the adjusted ORs and corresponding CIs for the subjects in second, third, and fourth quartiles were 1.48 (0.91–2.17), 2.42 (1.76–3.64), and 3.40 (2.11–5.29), respectively. The γ-radiation sensitivity was related to age and smoking status. In addition, a clear joint effect on cancer risk was found between γ-Radiation sensitivity and smoking status. The risk for ever smokers with high sensitivity was higher than those for never smokers with high sensitivity and ever smokers with low sensitivity (OR [CI], 4.67 [2.31–6.07] *vs.* 2.14 [1.40–3.06] vs. 2.42 [1.57–3.95], respectively). No significant interaction was found between both factors (*P* for interaction  = 0.42). We conclude that chromatid radiosensitivity is associated with gastric cancer susceptibility in a Chinese population.

## Introduction

Gastric cancer is one of the most frequent malignancies in China. Although more and more environmental risk factors [1], such as cigarette smoking, alcohol drinking, *Helicobacter pylori* (H. pylori) infection and excessive salt intake, have been identified, the genetic factors associated with sporadic gastric cancer remains to be mostly unclear. In past years, genotypic and phenotypic assays have been extensively used to study genetic variations in the general population and to investigate their possible associations with cancer risk [2], [3], [4]. Investigations using genotypic assays have shown that single nucleotide polymorphisms (SNP) and haplotypes related to genes encoding carcinogen-metabolizing enzymes and DNA repair proteins might be used to identify high-risk subgroups in the general population [5], [6]. With the advance of SNP chip technology, genome-wide association study has commonly been used to identify cancer susceptibility genes. A shared susceptibility locus in *PLCE1* at 10q23 for gastric adenocarcinoma and esophageal squamous cell carcinoma has been reported in ethnic Chinese subjects [7]. Meanwhile, investigations using phenotypic assays have helped to improve cancer risk assessment by elucidating complex genetic traits that might account for the net effects of several genetic pathways, for the cumulative effects of low-risk genetic variants, or simply for epigenetic alterations whose effects may be difficult to notice in genotypic studies. Of the two approaches, the phenotypic approach has the advantage of not depending on the discovery of new genes and has the potential for identifying individuals who harbor relevant germline mutations in as yet undiscovered genes.

To date, a variety of phenotype screening assays have been developed to assess cancer risk in population-based studies. Chief among them are assays that measure metaphase chromosomal aberrations [8], micronuclei [9], host cell reactivation [10], and mutagen-induced comet tails [11]. Another increasingly useful assay is the mutagen sensitivity assay (MSA) developed by Hsu *et al.* [12], [13]. This assay, which measures the number of mutagen-induced chromatid breaks per cell in cultured primary peripheral blood lymphocytes (PBLs) during the late S-G_2_ phase of the cell cycle, has been shown to provide useful biomarkers of susceptibility to different types of cancer including those of the lung [14], skin [15], head and neck [16], breast [17], liver [18], and brain [19].

Interindividual variations in the metabolism of carcinogens, susceptibility to chromosome damage in response to mutagens or carcinogens, and DNA repair capacity might contribute to variations in mutagen sensitivity among individuals. Also, it has been suggested that different mutagens may act via different molecular mechanisms and thereby activate different repair pathways. It follows that a person who is sensitive to one mutagen may be resistant to another. For this reason, several different challenge mutagens (e.g., γ-radiation, BPDE, and bleomycin) have come to be commonly used in performing MSAs. γ-Radiation is particular useful because it can cause oxidative damage and induce single- or double-strand breaks that are repaired by base excision and/or double-strand-break repair pathways. Moreover, γ-radiation-induced mutagen sensitivity of lymphocytes has been associated with an increased risk of breast cancer [17] and glioma [19]. In addition, Esther et al. conducted an inter-laboratory comparison to address the concerns of intra-observer, inter-observer, and inter-laboratory variations of mutagen sensitivity [20]. The correlation was high for all tests, suggesting a good concordance rate between different laboratories. As laboratory personnel of both laboratories were trained by using same technique, it is need to explore inter-laboratory concordance between different settings and countries.

Genotypic assays have identified many SNPs that are reportedly associated with increased or decreased gastric cancer risk [21]. However, few studies have attempted to determine gastric cancer risk phenotypically. Therefore, for the present study, we assembled a large hospital-based case-control population of 517 cases and 525 controls and used it to assess the relationship between γ-radiation-induced sensitivity and gastric cancer risk. To the best of our knowledge, this is the largest phenotypic study and the first γ-radiation sensitivity assay to assess gastric cancer risk.

## Subjects and Methods

### Ethics

This study was approved by the institutional review boards of the Fourth Military Medical University. Written informed consent with a signature was obtained from each patient.

### Study Population

In this case-control study, ethnicity of all participants was Chinese Han. All cases and controls were recruited without regard to age, sex or disease stage. A total of 517 incident cases who were newly diagnosed with histologically confirmed primary gastric adenocarcinoma were consecutively recruited from Department of General Surgery in Tangdu hospital affiliated to The Fourth Military Medical University, Xi’an, Shaanxi, China, between March 2008 and June 2011, which represented 81% of all new cases diagnosed at the same study period in Tangdu Hospital. All cases had no prior chemotherapy or radiotherapy. A cohort of 525 healthy controls having no prior history of cancer (except non-melanoma skin cancer) was simultaneously recruited from individuals who visited the same hospital for physical examination with a response rate of about 73% during the same time period as cases were recruited. Any case or control subject who had received a blood transfusion in the 6 months prior to enrollment was excluded from the study. After recruitment, cases and controls were frequency-matched by age (±5 years), sex, and the residential areas.

### Epidemiological Data

After signed informed consent was obtained from each individual, all participants were interviewed by trained staff interviewers by using a standardized epidemiological questionnaire. Each participant was required to provide detailed information on demographics, smoking history, alcohol consumption, dietary habits and family history of cancer after each interview, venous blood sample from each subject was drawn into coded tubes (3 mL into heparinized tube and 2 mL into regular tube) and forwarded for laboratory analysis. Laboratory personnel handling blood samples were blinded to the case-control status of each.

### Measurement of Serum Antibody IgG to *H. pylori*

The 2 mL of coagulated blood was centrifuged for 10 min at 400×g to collect the serum. The serum was then divided into three aliquots for storage in −80°C. H. pylori infection in all subjects was determined by *pylori* DTect test using a commercial IgG enzyme-linked immunosorbent assay kits (Diagnostic Technology, Pymble, Australia) according to the manufacture’s instruction. The test has been validated in Chinese populations with a high sensitivity and specificity for detection of H. pylori infection [22].

**Table 1:** Distribution of selected characteristics in gastric cancer cases and healthy controls.

Variables	Cases (n = 517)	Controls (n = 525)	*P* Value
**Sex, n (%)**			
Male	336 (64.9)	338 (64.4)	
Female	181 (35.1)	187 (35.6)	0.837
**Smoking Status**			
Never	229 (44.3)	330 (62.8)	
Ever	288 (55.7)	195 (37.2)	<0.001
**Alcohol Drinking**			
Never	252 (48.7)	344 (65.5)	
Ever	265 (51.3)	181 (34.5)	<0.001
**H. pylori Infection**			
Yes	317 (61.3)	265 (50.4)	
No	200 (38.7)	260 (49.6)	<0.001
**Age in years, n (%)**	52.9 (8.2)	53.2 (8.5)	0.562
**Pack-years** * **, mean (SD)**	44.8 (22.5)	32.4 (20.7)	<0.001
**Mutagen sensitivity** # **, mean (SD), b/c**	0.47 (0.20)	0.34 (0.17)	<0.001

SD: standard deviation.

* ever smokers only.

# Mutagen sensitivity was represented by number of chromatid breaks per cell (b/c).

### Mutagen Sensitivity Assay

A modified mutagen sensitivity assay as described previously by Cherry and Hsu [23], [24] was used in this study with γ-Radiation as the challenge mutagen. In brief, samples of fresh heparinized whole blood (1 mL each) were mixed with 9 mL of RPMI 1640 medium supplemented with 20% fetal bovine serum (Life Technologies, Inc., Gaithersburg, MD) and 112.5 µg/mL of phytohemagglutinin (Thermo Fisher Scientific, Remel Products, Lenexa, KS). Each mixture was incubated at 37°C with 5% CO_2_ for 70 hours to ensure the proliferation of PBLs and a good supply of mitotic cells for chromosome analysis. Subsequently, cells in one of the cultures were directly exposed to 1.5 Gy of γ-radiation from a ^60^Co irradiator (FJX Model, BINE High-Tech Co.,Ltd. Beijing, China) and then incubated for an additional 5 hours to allow time for DNA repair. Cells were then arrested in the mitotic stage by treatment with colcemid (Invitrogen) at a final concentration of 0.05 µg/mL for 1 hour before harvesting to induce mitotic arrest. The cells were harvested and prepared on slides as described previously [25]. After coding to ensure blinded evaluation, each slide was examined under a microscope, and the chromosome breaks in 50 well-spread metaphases were counted. Note that the decision to count breaks in only 50 metaphases was based on a previous study [26] showing that this conventional approach would provide adequate data. Only chromatid breaks were counted; chromatid gaps or attenuated regions were ignored. The mean number of chromatid breaks per cell was taken to represent the number of chromosome breaks in each sample. In this study, two well-trained scorers successively worked on the assessment of mutagen sensitivity by counting chromatid breaks in well-spread metaphases. Second scorer has been trained by first scorer until consistent result can be obtained on same slide when blinded to each other. Both scorers were blinded to case-control status.

**Table 2:** Gastric cancer risk as estimated by γ-radiation sensitivity.

Mutagen sensitivity#	Cases, n (%)	Controls, n (%)	Adjusted OR* (95%CI)
**By median** (50^th^ percentile)			
Low (<0.34 b/c)	172 (33)	262 (50)	1 (Reference)
High (≥0.34 b/c)	345 (67)	263 (50)	2.01(1.49–3.13)
**By quartile**			
1st	62 (12)	132 (25)	1 (Reference)
2nd	93 (18)	135 (26)	1.48(0.91–2.17)
3rd	150 (29)	128 (24)	2.42(1.76–3.64)
4th	212 (41)	130 (25)	3.40(2.11–5.29)
***P*** ** for trend**			<0.001

OR, odds ratio; CI, confidence interval.

# Mutagen sensitivity was represented by number of chromatid breaks per cell (b/c).

* Adjusted by age, sex, H. pylori infection, smoking and drinking status.

**Table 3:** Comparison of mutagen sensitivity among different subgroups in gastric cancer cases or controls.

	Cases				Controls		
	n		Mean (SD), b/c	*p* a	n	Mean (SD), b/c	*p* a
**Sex**							
Male	336		0.47 (0.19)		338	0.33 (0.16)	
Female	181		0.47 (0.21)	1.000	187	0.35 (0.18)	0.190
**Age**							
<53	267	0.49 (0.22)			260	0.33 (0.17)	
≥53	250	0.45 (0.17)		0.022	265	0.35 (0.17)	0.178
**Smoking status**							
Never	229	0.43 (0.19)			330	0.31 (0.15)	
Ever	288	0.50 (0.22)		<0.001	195	0.38 (0.19)	<0.001
**Alcohol drinking**							
Never	252	0.47 (0.18)			344	0.34 (0.18)	
Ever	265	0.47 (0.21)		1.000	181	0.34 (0.16)	1.000
**H. pylori infection**							
Yes	317	0.48 (0.20)			265	0.34 (0.17)	
No	200	0.46 (0.19)		0.260	260	0.34 (0.17)	1.000

SD: standard deviation.

a *p* values were determined by Student’s *t* test to assess the difference of mutagen sensitivity between two different subgroups in cases or controls.

Mutagen sensitivity was represented by number of chromatid breaks per cell (b/c).

### Statistical Analysis

All statistical analyses were performed using the statistical package SPSS 18.0 for Microsoft Windows (SPSS, Chicago, IL). Smoking and drinking status were categorized as dichotomized variables. Individuals who had smoked less than 100 cigarettes in his or her lifetime were defined as never smokers, and those that consumed 3 and more standard cups of alcohol each week for over 6 months were considered as ever drinkers. Pack-years were defined as the mean number of cigarettes smoked per day divided by 20 and then multiplied by smoking years. Pearson’s χ^2^ test was used to examine differences in the distribution of cases and controls in terms of sex, smoking and drinking status. Student’s *t* test was used to analyze normally distributed continuous variables, such as age, cigarette pack-years (among ever smokers) and γ-radiation sensitivity. In addition, subjects were dichotomized according to γ-radiation sensitivity by using the 50^th^ percentile value for the controls as the cutoff point or stratified into quartiles based on the number of chromatid breaks per cell in the controls for further analyses as a categorical variable. An individual was considered sensitive to γ-radiation if the number of chromatid breaks per cell was equal to or larger than the 50^th^ percentile value for the controls. Subjects were dichotomized by age according to the median age of the controls. The association between the number of chromatid breaks per cell and gastric cancer risk was estimated by calculating and comparing adjusted ORs and their corresponding CIs. To account for the potentially confounding effects of age, sex, smoking status, drinking status and H. pylori infection, unconditional logistic regression analysis with multiple covariates was performed. Stratified analyses were performed to compare γ-radiation sensitivity among different subgroups of cases or controls, to assess the risk of gastric cancer associated with γ-radiation sensitivity in those subgroups, and to evaluate the joint effect of γ-radiation sensitivity and smoking status on gastric cancer risk. All *P* values were based on two-sided tests. A probability level of 0.05 was used as the criterion for statistical significance.

**Table 4:** Joint effect of mutagen sensitivity and smoking in gastric cancer risk.

Mutagen sensitivity#	Smoking status	Cases n (%)	Controls n (%)	Adjusted OR* (95%CI)
Low	Never smoker	73 (14)	165 (31)	1 (Reference)
High	Never smoker	156 (30)	165 (31)	2.14 (1.40–3.06)
Low	Ever smoker	98 (19)	92 (18)	2.42 (1.57–3.95)
High *P* for interaction	Ever smoker	190 (37)	103 (20)	4.67 (2.31–6.07)0.42

* Adjusted for age, sex, H. pylori infection and drinking status.

# Mutagen sensitivity was represented by number of chromatid breaks per cell (b/c).

## Results

The characteristics of the 517 cases and 525 controls are summarized in Table 1. The cases and healthy controls were well-matched in terms of sex distribution (*P* = 0.837) and mean age (52.9±8.2 *vs.* 53.2±8.5 years old, *P* = 0.562). Compared with the controls, the cases had proportionally more ever smokers (55.7% *vs.* 37.2%) and proportionally fewer never smokers (44.3% *vs.* 62.8%) and smoked more heavily (Mean [SD] pack-years, 44.8 [22.5] *vs.* 32.4 [20.7]; *P*<0.001). As expected, cases had more ever drinkers then controls (51.3% *vs.* 34.5%, *P*<0.001). Cases had a significantly higher percentage of H. pylori infection than controls (61.3% *vs.* 50.4%; p<0.001).

The γ-Radiation -induced chromatid breaks per cell were significantly more frequent in cases than in controls (mean [SD], 0.47 [0.20] *vs.* 0.34 [0.17]; *P*<0.001). After dichotomization by radiation sensitivity at the 50^th^ percentile cutoff for controls (0.34 breaks per cell), radiation-sensitive individuals were found to be at significantly greater risk for gastric cancer than non-sensitive individuals (OR [95%CI], 2.01 [1.49–3.13]) after adjustment for age, sex, H. pylori infection, smoking and drinking status (Table 2). Subsequent stratification by quartiles as described above in Methods revealed a statistically significant dose-response relationship between the number of breaks per cell and gastric cancer risk (*P*<0.001). The adjusted ORs and corresponding CIs for the second, third, and fourth quartiles were 1.48 (0.91–2.17), 2.42 (1.76–3.64), and 3.40 (2.11–5.29), respectively (Table 2). As indicated in Figure S1, a large overlap of MSA data was noted between cases and control. Receiver operating characteristic (ROC) analysis indicated a best cut-off value of 0.475 for a risk biomarker with positive and negative predictive values of 0.499 and 0.829, respectively. These data suggest a limited separate use of γ-radiation sensitivity as a biomarker to predict gastric cancer risk.

The γ-radiation sensitivity profiles within the case and control groups were compared in terms of sex, age, smoking use, drinking status, and H. pylori infection (Table 3). The statistically significant difference noted was an association between age and γ-radiation-induced sensitivity in the case group and an association between smoking statuses. In brief, case subjects <53 years old were significantly more sensitive to γ-radiation than were those ≥53 years old (mean [SD] chromatid breaks per cell, 0.49 [0.22] *vs.* 0.45 [0.17]; *P* = 0.022). Ever smokers were significantly more sensitive than never smokers in both cases (0.50 [0.22] *vs.* 0.43 [0.19]; *P*<0.001) and controls (0.38 [0.19] *vs.* 0.31 [0.15]; *P*<0.001).

As revealed by stratified analyses (Table S1), cases exhibited no significant difference on the increased risk (OR [95%CI]) associated with γ-radiation sensitivity in different subgroups, such as in females and in males (2.65 [1.61–4.24] *vs.* 1.88 [1.38–2.72]), in old persons and in younger persons (2.72 [1.78–4.38] *vs.* 1.83 [1.14–2.91], in never smokers and in ever smokers (2.14 [1.40–3.06] *vs.* 1.73 [1.25–2.91], in never drinkers and in ever drinkers (2.25 [1.53–3.17] *vs.* 1.86 [1.29–2.95] as well as in individuals without H. pylori infection and in those with H. pylori infection (2.56 [1.50–4.06] *vs.* 1.84 [1.27–2.85]. A joint effect on cancer risk was found between γ-Radiation sensitivity and smoking status by comparing the risks for sensitive ever smokers, sensitive never smokers, and non-sensitive smokers against the risk for non-sensitive never smokers (i.e., the reference group) (Table 4). In brief, the risk for ever smokers with high sensitivity was higher than those for never smokers with high sensitivity and ever smokers with low sensitivity (OR [CI], 4.67 [2.31–6.07] *vs.* 2.14 [1.40–3.06] *vs.* 2.42 [1.57–3.95], respectively, *P* for interaction  = 0.42).

## Discussion

In a large case-control population, we have demonstrated that genetic instability (measured as sensitivity to radiation-induced chromatid breaks) is associated with an increased risk of gastric cancer. Our results are in line with those of previous studies showing γ-radiation sensitivity to be an independent risk factor for breast cancer [17], [27], [28] and glioma [19].

As an integrated phenotypic biomarker, mutagen sensitivity potentially represents the endpoint of many different pathways by which a cell may suffer and repair DNA damage in response to mutagen challenge [29]. The mechanism underlying susceptibility to chromosome damage, however, remains unclear in many respects. Gajecka *et al.* [30] have suggested that the chromatid breaks induced by mutagen challenge in vitro do not occur at random sites, but that in most cases they occur in regions containing loci involved in DNA repair and cell cycle regulation, suppressor genes, and oncogenes. Pandita and Hittelman [31] have suggested that the mutagen sensitivity phenotype may reflect inherent structural alterations in chromatin that increase the chances of DNA damage being translated into chromosome damage. Hsu [32] has suggested that susceptibility to chromosome damage varies along a continuum, at the extreme end of which lies recognized chromosome fragility syndromes such as Fanconi’s anemia or ataxia-telangiectasia. Moreover, the apparently broad effect of mutagen sensitivity on diverse cancers suggests that multiple genes in various DNA repair pathways may contribute to this phenotype. When Wei *et al*. [33] evaluated DNA repair activity in 16 established cell lines by conducting host cell reactivation and mutagen sensitivity assays in parallel, they found a statistically significant association between decreased DNA repair activity and higher rates of mutagen-induced chromatid breaks. This suggests that repair fidelity may be hampered in individuals who are hypersensitive to mutagens. Berwick and Vineis [34] have suggested that the mutagen sensitivity assay indirectly measures general and nonspecific impairment of the DNA repair machinery. Our present findings provide additional support for the notion that defective DNA repair is associated with increased gastric cancer risk.

Although γ-radiation sensitivity in our study population was clearly not affected by sex, drinking status, or H. pylori infection, which is consistent with previous reports [35], [36], [37], it did appear to be affected by age and smoking status. Indeed, we observed that the frequency of chromatid breaks per cell among case subjects was higher for the younger ones (i.e., those less than 53 years old). The increasing radiosensitivity with decreasing age suggests that genetic variation is more important for a younger age group whereas environmental risk factors are more important with increasing age, which is consistent with expectations. Previous observation by Hsu *et al.* [38] also demonstrates that mutagen sensitivity tends to decrease with increasing age in heavy smokers over 50 years old. Meanwhile, our ability to link smoking status to mutagen sensitivity suggests that smoking may be responsible for the differences in susceptibility we observed between groups. In addition, we compared the level of γ-radiation sensitivity (measured as breaks per cell, b/c) in control subjects among different studies and found notable difference by population. In comparison to b/c values in our study (Mean ±SD, 0.34±0.17), Wang et al. [28] have reported obviously higher b/c values (0.44±0.16) for controls, while Natarajan et al. [27] have reported obviously lower b/c values (0.24±0.12). The variation between studies possibly results from some differences in assay procedure, radiation dose, and scoring criteria. Future efforts should be directed at setting up a uniform b/c level for each mutagen to facilitate inter-study comparisons and potential pooled analyses.

Despite the promising findings, our study has several potential limitations. First, it may be argued that cancer patients simply suffer more chromosome breaks in vivo and thus only appear to exhibit more breaks after γ-radiation treatment. Previous studies [38], [39] have extensively analyzed the frequency of baseline “spontaneous” breaks in vitro and found them to be extremely rare (±0.02 breaks per cell) in both patients and healthy controls. Thus, we do not routinely report such baseline breaks separately.

Second, it may be argued that the mutagen sensitivity of PBLs does not accurately reflect the mutagen sensitivity of target tissue. However, there is ample evidence to the contrary. Cheng *et al.* [40] established that PHA-stimulated lymphocytes might be used as a tissue surrogate in estimating DNA repair capacity by demonstrating the similar expression of several DNA repair genes in PHA-stimulated lymphocytes, skin, breast, liver, and prostate. Seetharam *et al.* [41] demonstrated that UV-irradiated plasmids replicated in XP lymphoblasts and XP fibroblasts suffered very similar types of mutations, thus implying that different cell types from the same individual may exhibit similar mutagenic damage. In a study of individuals with precancerous disease, Udumudi *et al.* [42] detected genetic instability not only in the cervical epithelial cells but also in the PBLs of their subjects. Together, these findings suggest that the mutagen sensitivity of lymphocytes does indeed reflect the repair capacity of a donor’s target tissue.

A third concern, directly attributable to the case-control design of our study, is that mutagen sensitivity may be an effect rather than a cause of gastric cancer. However, there is mounting evidence that increased susceptibility to induced chromatid breaks does have a genetic basis. Patel *et al.* [43] reported that first-degree relatives of breast cancer patients had more radiation-induced chromosome breaks than did controls. In a cohort study of 3182 workers exposed occupationally to mutagenic agents and studied for chromosomal aberrations at baseline, Hagmar *et al.* [44] noted a statistically significant linear trend toward increased cancer risk with increasing number of aberrations. In a study of twins [45], authors found strong, direct evidence that mutagen sensitivity is highly heritable, thereby validating the use of mutagen sensitivity as a marker of cancer susceptibility. In addition, a prospective analysis by Chao et al. supports the hypothesis that sensitivity to mutagens increases the risk of neoplastic progression in persons with Barrett’s esophagus, particularly those with 17p LOH including TP53 [46].

In summary, our data indicate that increased sensitivity to γ-radiation is associated with an increased risk of developing gastric cancer. To our knowledge, this study is the first published case-control study to date to address the role of increased mutagen sensitivity in gastric tumorigenesis. Our present findings warrant future studies aimed at identifying the genes responsible for the mutagen sensitivity phenotype and elucidating the molecular mechanisms underlying variations in mutagen sensitivity between individuals. In addition, we can expected that, when combined with other risk factors, γ-radiation sensitivity will contribute to build a risk prediction model for gastric cancer.

## Supporting Information

Figure S1 **Distribution of mutagen sensitivity data for cases versus controls.**
(DOC)

Table S1 **Estimates of GC risk associated with mutagen sensitivity stratified by selected variables.**
(DOC)
