# Supplementary figures and images for: High γ-Radiation Sensitivity Is Associated with Increased Gastric Cancer Risk in a Chinese Han Population: A Case-Control Analysis
Source: PLoS One. 2012 Aug 22;7(8):e43625. doi: 10.1371/journal.pone.0043625 (PMC3425539; doi:10.1371/journal.pone.0043625)

**Figure legend.**

**Figure S1.** Distribution of mutagen sensitivity data for cases versus controls


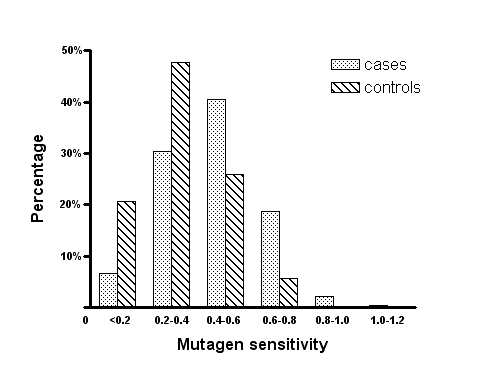

Supplement: Figure S1 — Distribution of mutagen sensitivity data for cases versus controls. (DOC) [file pone.0043625.s001.doc]
