# Supplementary material for: High γ-Radiation Sensitivity Is Associated with Increased Gastric Cancer Risk in a Chinese Han Population: A Case-Control Analysis
Source: PLoS One. 2012 Aug 22;7(8):e43625. doi: 10.1371/journal.pone.0043625 (PMC3425539; doi:10.1371/journal.pone.0043625)
Supplement: Table S1 — Estimates of GC risk associated with mutagen sensitivity stratified by selected variables. (DOC) [file pone.0043625.s002.doc]

**Table S1. Estimates of GC risk associated with mutagen sensitivity** stratified by selected variables

| **Variables Cases, n (%) Controls, n (%) OR (95% CI)^a^** | | | | |
| --- | --- | --- | --- | --- |
| **Sex** |  |  |  |  |
| Male |  |  |  |  |
| Low (<0.34 b/c) | 111 (33) | 166 (49) | 1 (Reference) |  |
| High (≥0.34 b/c) | 225 (67) | 172 (51) | 1.88（1.38-2.72） |  |
| Female |  |  |  |  |
| Low (<0.34 b/c) | 52 (29) | 97 (52) | 1 (Reference) |  |
| High (≥0.34 b/c) | 129 (71) | 90 (48) | 2.65（1.61-4.24） |  |
| **Age (in years)** |  |  |  |  |
| <53 |  |  |  |  |
| Low (<0.34 b/c) | 99 (37) | 136 (52) | 1 (Reference) |  |
| High (≥0.34 b/c) | 168 (63) | 124 (48) | 1.83（1.14-2.91） |  |
| ≥ 53 |  |  |  |  |
| Low (<0.34 b/c) | 65 (26) | 130 (49) | 1 (Reference) |  |
| High (≥0.34 b/c) | 185 (74) | 135 (51) | 2.72（1.78-4.38） |  |
| **Smoking status** |  |  |  |  |
| Never |  |  |  |  |
| Low (<0.34 b/c) | 73 (32) | 165 (50) | 1 (Reference) |  |
| High (≥0.34 b/c) | 156 (68) | 165 (50) | 2.14（1.40-3.06） |  |
| Ever |  |  |  |  |
| Low (<0.34 b/c) | 98 (34) | 92 (47) | 1 (Reference) |  |
| High (≥0.34 b/c) | 190 (66) | 103 (53) | 1.73（1.25-2.91） |  |
| **Drinking status** |  |  |  |  |
| Never |  |  |  |  |
| Low (<0.34 b/c) | 76 (30) | 169 (49) | 1 (Reference) |  |
| High (≥0.34 b/c) | 176 (70) | 175 (51) | 2.25（1.53-3.17） |  |
| Ever |  |  |  |  |
| Low (<0.34 b/c) | 82 (31) | 83 (46) | 1 (Reference) |  |
| High (≥0.34 b/c) | 183 (69) | 98 (54) | 1.86（1.29-2.95） |  |
| **H. pylori infection** |  |  |  |  |
| Yes |  |  |  |  |
| Low (<0.34 b/c) | 105 (33) | 127 (48) | 1 (Reference) |  |
| High (≥0.34 b/c) | 212 (67) | 138 (52) | 1.84（1.27-2.85） |  |
| No |  |  |  |  |
| Low (<0.34 b/c) | 56 (28) | 133 (51) | 1 (Reference) |  |
| High (≥0.34 b/c) | 144 (72) | 127 (49) | 2.56（1.50-4.06） |  |

^a^Adjusted for age, sex, H. pylori infection, smoking and drinking status, where appropriate.

Mutagen sensitivity was represented by number of chromatid breaks per cell (b/c).
